# RETRACTION: ZC3H15 Correlates with a Poor Prognosis and Tumor Progression in Melanoma

**DOI:** 10.1155/bmri/9853504

**Published:** 2026-01-13

**Authors:** BioMed Research International

RETRACTION: Q. Li, J. Hou, C. Guo, et al., “ZC3H15 Correlates with a Poor Prognosis and Tumor Progression in Melanoma,” *BioMed Research International* 2021, no. 1 (2021): 8305299, https://doi.org/10.1155/2021/8305299.

The above article, published online on 27 December 2021 in Wiley Online Library (http://wileyonlinelibrary.com), has been retracted by agreement between the editorial board and John Wiley & Sons Ltd. The retraction has been agreed following an investigation of the concerns identified with the western blots presented in Figures 1, 2 and 4.

More specifically:

• In Figure 1e, the Tubulin panel appears to be duplicated with Figure 1C of [1] with a vertical stretch.
• In Figure 2D the Tubulin panel appears to be duplicated with Figure 5C of [1] with horizontal mirroring.
• Within Figure 4, several western blot panels appear to be duplicated.

As a result of the investigation, the data and conclusions of this article are considered unreliable.

The authors agree to the retraction.
